# Supplementary material for: Between extreme simplification and ideal optimization: antennal sensilla morphology of miniaturized Megaphragma wasps (Hymenoptera: Trichogrammatidae)
Source: PeerJ. 2018 Nov 30;6:e6005. doi: 10.7717/peerj.6005 (PMC6276593; doi:10.7717/peerj.6005)
Supplement: Table S1 [file peerj-06-6005-s001.docx]

**Table S1.** Antennae length in *M. mymaripenne, M. amalphitanum* and *M. caribea* males and females.

| **Species, sex** | **Antennae length,**  **mean ± sd, µm** |
| --- | --- |
| *M. mymaripenne*, female | 140±9 (n=10) |
| *M. amalphitanum*, female | 144±9 (n=10) |
| *M. amalphitanum*, male | 136±10 (n=10) |
| *M. caribea*, female | 120±7 (n=6) |
| *M. caribea*, male | 120±9 (n=6) |
