# Supplementary material for: Between extreme simplification and ideal optimization: antennal sensilla morphology of miniaturized Megaphragma wasps (Hymenoptera: Trichogrammatidae)
Source: PeerJ. 2018 Nov 30;6:e6005. doi: 10.7717/peerj.6005 (PMC6276593; doi:10.7717/peerj.6005)
Supplement: Table S2 — Blue color indicates interspecies comparisons, yellow –intersex comparisons, green color stands for comparisons of the specimens of the same sex and species. «*» sign indicates significant difference (p-value < 0.05). Sc = scape, pd = pedicel, fl 1 = 1st flagellomere, fl 2 = 2nd flagellomere, fl 1+2 = joint 1st and 2nd flagellomere, fl 3 = 3d flagellomere; M. m. = Megaphragma mymaripenne, M. a., f = M. amalphitanum, female, M. a., m = M. amalphitanum, male, M. c., f = M. caribea, female, M. c., m = M. caribea, male. [file peerj-06-6005-s002.docx]

**Table S2** Significance of the differences among group means of the lengths (upper half of the table) and diameters (bottom half of the table) of sensillum type calculated with ANOVA. Blue color indicates interspecies comparisons, yellow – intersex comparisons, green color stands for comparisons of the specimens of the same sex and species. «*» sign indicates significant difference (p-value<0.05). Sc = scape, pd = pedicel, fl 1 = 1^st^ flagellomere, fl 2 = 2^nd^ flagellomere, fl 1+2 = joint 1^st^ and 2^nd^ flagellomere, fl 3 = 3^d^ flagellomere; M. m. = *Megaphragma mymaripenne*, M. a., f = *M. amalphitanum*, female, M. a., m = *M. amalphitanum*, male, M. c., f = *M. caribea*, female, M. c., m = *M. caribea*, male.

Sensilla chaetica, aporous (ChS-AP)

|  |  | Sp., sex | M. m. | | | M. a., f | | | M. a., m | | | M. c., f | | M. c., m | |  |
| --- | --- | --- | --- | --- | --- | --- | --- | --- | --- | --- | --- | --- | --- | --- | --- | --- |
|  | Sp., sex | Loc. | sc | pd | fl 1 | sc | pd | fl 1 | sc | pd | fl 1 | sc | pd | sc | pd |  |
|  | M. m. | sc |  | - | - | - |  |  |  |  |  | * |  |  |  |  |
|  |  | pd | - |  | * |  | * |  |  |  |  |  | * |  |  |  |
|  |  | fl 1 | - | - |  |  |  | * |  |  |  |  |  |  |  |  |
|  | M. a., f | sc | - |  |  |  | - | * | - |  |  | * |  |  |  |  |
|  |  | pd |  | - |  | - |  | * |  | - |  |  | * |  |  |  |
|  |  | fl 1 |  |  | - | - | - |  |  |  | * |  |  |  |  |  |
|  | M. a., m | sc |  |  |  | * |  |  |  | * | * |  |  | * |  |  |
|  |  | pd |  |  |  |  | * |  | * |  | - |  |  |  | * |  |
|  |  | fl 1 |  |  |  |  |  | * | - | - |  |  |  |  |  |  |
|  | M. c., f | sc |  | * |  | - |  |  |  |  |  |  | - | - |  |  |
|  |  | pd | * |  |  |  | - |  |  |  |  | - |  |  | * |  |
|  | M. c., m | sc |  |  |  |  |  |  | - |  |  | * |  |  | * |  |
|  |  | pd |  |  |  |  |  |  |  | - |  |  | - | - |  |  |

Sensilla trichodea, aporous type 1 (TS1-AP)

|  | Sp., sex | M. m. | | | M. a., f | | | M. a., m | | M. c., f | | M. c., m | |
| --- | --- | --- | --- | --- | --- | --- | --- | --- | --- | --- | --- | --- | --- |
| Sp., sex | Loc. | fl 1 | fl 2 | fl 3 | fl 1 | fl 2 | fl 3 | fl 1 | fl 2 | fl 1+2 | fl 3 | fl 1+2 | fl 3 |
| M. m. | fl 1 |  | * | * | - |  |  |  |  | * |  |  |  |
|  | fl 2 | * |  | * |  | * |  |  |  | * |  |  |  |
|  | fl 3 | * | * |  |  |  | - |  |  |  | * |  |  |
| M. a., f | fl 1 | - |  |  |  | * | * | * |  | - |  |  |  |
|  | fl 2 |  | * |  | - |  | * |  | * | * |  |  |  |
|  | fl 3 |  |  | - | * | * |  |  |  |  | * |  |  |
| M. a., m | fl 1 |  |  |  | * |  |  |  | * |  |  | * |  |
|  | fl 2 |  |  |  |  | * |  | - |  |  |  | * |  |
| M. c., f | fl 1+2 | * | * |  | * | * |  |  |  |  | * | * |  |
|  | fl 3 |  |  | * |  |  | * |  |  | * |  |  | * |
| M. c., m | fl 1+2 |  |  |  |  |  |  | - | - | * |  |  | * |
|  | fl 3 |  |  |  |  |  |  |  |  |  | * | * |  |

Sensilla styloconica (SS)

|  | Sp., sex | M. m. | | M. a., f | M. a., m | M. c., f | | | | M. c., m | |  |  |
| --- | --- | --- | --- | --- | --- | --- | --- | --- | --- | --- | --- | --- | --- |
| Sp., sex | Loc. | fl 2 | fl 3 | fl 3 | fl 3 | fl 1+2 | | fl 3 | | fl1+2 | fl 3 |  |  |
| M. m. | fl 2 |  | * |  |  | * | |  | |  |  |  |  |
|  | fl 3 | - |  | * |  |  | | - | |  |  |  |  |
| M. a., f | fl 3 |  | - |  | * | |  | | * |  |  |  | |
| M. a., m | fl 3 |  |  | * |  | |  | |  | - |  |  | |
| M. c., f | fl 1+2 | * |  |  |  |  | | * | | - |  |  |  |
|  | fl 3 |  | - | - |  | - | |  | |  | - |  |  |
| M. c., m | fl 1+2 |  |  |  |  | - | |  | |  | - |  |  |
|  | fl 3 |  |  |  | * |  | | - | | - |  |  |  |
|  |  |  |  |  |  |  | |  | |  |  |  |  |

Sensilla trichodea, aporous type 2 (TS2-AP)

|  | Sp., sex | M. m. | | M. a., f | | M. a., m | | M. c., f | M. c., m |
| --- | --- | --- | --- | --- | --- | --- | --- | --- | --- |
| Sp., sex | Loc. | sc | pd | sc | pd | sc | pd | sc | sc |
| M. m. | sc |  | * | - |  |  |  | - |  |
|  | pd | - |  |  | - |  |  |  |  |
| M. a., f | sc | * |  |  | * | * |  | * |  |
|  | pd |  | - | * |  |  | - |  |  |
| M. a., m | sc |  |  | - |  |  | * |  | * |
|  | pd |  |  |  | - | - |  |  |  |
| M. c., f | sc | - |  | - |  |  |  |  | - |
| M. c., m | sc |  |  |  |  | - |  | - |  |

Multiporous placoid sensilla (MPS) Sensilla trichodea, uniporous (TS-UP)

|  |  | | |  | |  | |  |  |  |
| --- | --- | --- | --- | --- | --- | --- | --- | --- | --- | --- |
| Sp., sex | | M. m., f | | | M. a. f | | M. a. m | | M. c., f | M. c., m |
| M. m., f | | |  | | - | |  | | - |  |
| M. a. f | | | - | |  | | - | | - |  |
| M. a. m | | |  | | - | |  | |  | * |
| M. c., f | | | - | | - | |  | |  | * |
| M. c., m | | |  | |  | | - | | - |  |

| Sp., sex | M. m., f | | M. a. f | | M. a. m | | M. c., f | | M. c., m | |
| --- | --- | --- | --- | --- | --- | --- | --- | --- | --- | --- |
| M. m., f | |  | | - | |  | | - | |  |
| M. a. f | - | |  | | * | | * | |  | |
| M. a. m |  | | * | |  | |  | | - | |
| M. c., f | - | | - | |  | |  | | - | |
| M. c., m |  | |  | | - | | - | |  | |

Sensilla basiconica (BS) Placoid sensilla (PS)

|  |  | |  |  |  |  |
| --- | --- | --- | --- | --- | --- | --- |
| Sp., sex | | M. m., f | | M. a., f | M. c., f |  |
| M. m., f | |  | | - | - |  |
| M. a., f | | - | |  | * |  |
| M. c., f | | * | | * |  |  |

|  |  |  |  |
| --- | --- | --- | --- |
| Sp., sex | M. m., f | M. a., f | M. c., f |
| M. m., f |  | - | - |
| M. a., f | * |  | * |
| M. c., f | - | * |  |

|  |  |  |
| --- | --- | --- |
