# Supplementary material for: Between extreme simplification and ideal optimization: antennal sensilla morphology of miniaturized Megaphragma wasps (Hymenoptera: Trichogrammatidae)
Source: PeerJ. 2018 Nov 30;6:e6005. doi: 10.7717/peerj.6005 (PMC6276593; doi:10.7717/peerj.6005)
Supplement: Table S3 — Sc = scape, rad = radicula, pd = pedicel, an = anelli, fl 1 = 1st flagellomere, fl 2 = 2nd flagellomere, fl 1+2 = joint 1st and 2nd flagellomere, fl 3 = 3rd flagellomere. Mean ± SD for length and diameter is given. Measurements unit - µm. n—number of measured sensilla. [file peerj-06-6005-s003.docx]

**Table S3.** *Megaphragma* antennal sensilla sizes. Sc = scape, rad = radicula, pd = pedicel, an = anelli, fl 1 = 1^st^ flagellomere, fl 2 = 2^nd^ flagellomere, fl 1+2 = joint 1^st^ and 2^nd^ flagellomere, fl 3 = 3^rd^ flagellomere. Mean ± SD for length and diameter is given. Measurements unit - μm. n - number of measured sensilla.

| Sensillum type | Localisation | *Megaphragma amalphitanum* | | | | | | | | | |
| --- | --- | --- | --- | --- | --- | --- | --- | --- | --- | --- | --- |
|  |  | Female | | | | | Male | | | | |
|  |  | Length | Min length | Diameter | Min diameter | n | Length | Min length | Diameter | Min diameter | n |
| ChS-AP | sc | 10.8±2.23 | 6.99 | 0.92±0.16 | 0.71 | 10 | 12.3±3.08 | 7.38 | 0.76±0.11 | 0.60 | 10 |
|  | pd | 10.2±2.04 | 6.99 | 0.86±0.17 | 0.65 | 11 | 8.79±1.94 | 7.12 | 0.65±0.09 | 0.53 | 10 |
|  | fl 1 | 13.7±2.33 | 9.93 | 0.93±0.16 | 0.69 | 10 | 8.57±1.95 | 6.01 | 0.72±0.11 | 0.52 | 10 |
|  | all | 11.5±2.62 | 6.99 | 0.9±0.16 | 0.65 | 31 | 9.89±2.88 | 6.01 | 0.71±0.11 | 0.52 | 30 |
| TS1-AP | fl 1 | 9.36±1.55 | 5.35 | 0.8±0.07 | 0.66 | 23 | 6.82±1.35 | 4.45 | 0.68±0.1 | 0.53 | 12 |
|  | fl 2 | 11.6±2.99 | 6.19 | 0.78±0.1 | 0.56 | 70 | 7.79±1.6 | 5.03 | 0.65±0.09 | 0.52 | 60 |
|  | fl 3 | 22.5±6.47 | 13.71 | 1.01±0.2 | 0.64 | 16 | - | - | - | - | - |
|  | all | 12.7±5.42 | 5.35 | 0.82±0.14 | 0.56 | 109 | 7.63±1.59 | 4.45 | 0.65±0.09 | 0.52 | 72 |
| TS-UP | fl 2 | 37.1±5.59 | 10.91 | 1.36±0.23 | 0.88 | 14 | - | - | - | - | - |
|  | fl 3 | - | - | - | - | - | 33.6±4.46 | 22.00 | 1.34±0.16 | 0.93 | 21 |
|  | all | 37.1±5.59 | 10.91 | 1.36±0.23 | 0.88 | 14 | 33.6±4.46 | 22.00 | 1.34±0.16 | 0.93 | 21 |
| SS | fl 2 | - | - | - | - | - | - | - | - | - | - |
|  | fl 3 | 7±0.72 | 5.65 | 0.93±0.11 | 0.81 | 10 | 5.05±0.48 | 4.46 | 0.76±0.09 | 0.63 | 10 |
|  | all | 7±0.72 | 5.65 | 0.93±0.11 | 0.81 | 10 | 5.05±0.48 | 4.46 | 0.76±0.09 | 0.63 | 10 |
| TS2-AP | sc | 1.06±0.11 | 0.88 | 0.62±0.13 | 0.49 | 11 | 1.31±0.26 | 1.01 | 0.62±0.05 | 0.54 | 10 |
|  | pd | 0.68±0.09 | 0.48 | 0.47±0.12 | 0.32 | 10 | 0.74±0.18 | 0.50 | 0.44±0.09 | 0.29 | 10 |
|  | all | 0.88±0.22 | 0.48 | 0.55±0.14 | 0.32 | 21 | 1.03±0.36 | 0.50 | 0.53±0.12 | 0.29 | 20 |
| BS | fl 3 | 17.1±2.04 | 8.23 | 1.96±0.19 | 1.71 | 10 | - | - | - | - | - |
| PS | fl 3 | 39.9±4.15 | 12.11 | 2.48±0.6 | 1.68 | 19 | - | - | - | - | - |
| PS, tip | fl 3 | 11.7±4.32 | 5.31 | 2.48±0.6 | 1.69 | 19 | - | - | - | - | - |
| MPS | fl 3 | 37.3±3.58 | 16.42 | 2.62±0.27 | 2.12 | 10 | 21.9±5.61 | 20.39 | 1.82±0.38 | 1.11 | 16 |
| MPS, tip | fl 3 | 3.69±1.36 | 1.93 | 2.62±0.27 | 2.12 | 10 | 3.76±4.51 | 1.11 | 1.8±0.39 | 1.11 | 15 |

| Sensillum type | Localisation | *Megaphragma caribea* | | | | | | | | | |
| --- | --- | --- | --- | --- | --- | --- | --- | --- | --- | --- | --- |
|  |  | Female | | | | | Male | | | | |
|  |  | Length | Min length | Diameter | Min diameter | n | Length | Min length | Diameter | Min diameter | n |
| ChS-AP | sc | 8.06±2.07 | 5.35 | 0.78±0.12 | 0.60 | 11 | 6.91±0.94 | 5.51 | 0.62±0.07 | 0.52 | 9 |
|  | pd | 8.03±1.15 | 6.61 | 0.73±0.13 | 0.45 | 22 | 5.9±0.83 | 4.01 | 0.7±0.14 | 0.41 | 15 |
|  | fl 1+2 | - | - | - | - | - | - | - | - | - | - |
|  | all | 8.04±1.49 | 5.35 | 0.75±0.12 | 0.45 | 33 | 6.28±0.99 | 4.01 | 0.67±0.13 | 0.41 | 24 |
| TS1-AP | fl 1+2 | 7.42±1.86 | 4.55 | 0.68±0.09 | 0.45 | 33 | 5.05±1.26 | 2.43 | 0.62±0.11 | 0.41 | 101 |
|  |  |  |  |  |  |  |  |  |  |  |  |
|  | fl 3 | 11.36±5.48 | 6.31 | 0.83±0.16 | 0.63 | 11 | 6.03±1.2 | 3.37 | 0.68±0.16 | 0.46 | 13 |
|  | all | 8.41±3.54 | 4.55 | 0.72±0.13 | 0.45 | 44 | 5.16±1.29 | 2.43 | 0.62±0.12 | 0.41 | 114 |
| TS-UP | fl 1+2 | 33.03±5.97 | 24.06 | 1.19±0.12 | 1.01 | 12 | - | - | - | - | - |
|  | fl 3 | - | - | - | - | - | 20.42±2.87 | 14.52 | 1.28±0.2 | 1.02 | 21 |
|  | all | 33.03±5.97 | 24.06 | 1.19±0.12 | 1.01 | 12 | 20.42±2.87 | 14.52 | 1.28±0.2 | 1.02 | 21 |
| SS | fl 1+2 | 4.01±0.65 | 3.31 | 1.06±0.03 | 1.03 | 3 | 4.45±0.98 | 3.30 | 1.01±0.16 | 0.73 | 8 |
|  | fl 3 | 5.23±0.54 | 4.37 | 0.94±0.11 | 0.83 | 5 | 4.74±1.54 | 2.89 | 0.98±0.16 | 0.88 | 6 |
|  | all | 4.78±0.83 | 3.31 | 0.99±0.11 | 0.83 | 8 | 4.58±1.2 | 2.89 | 1±0.15 | 0.73 | 14 |
| TS2-AP | sc | 0.89±0.21 | 0.60 | 0.57±0.03 | 0.52 | 6 | 0.97±0.23 | 0.50 | 0.61±0.27 | 0.39 | 17 |
|  | pd | n/c | n/c | n/c | n/c | n/c | n/c | n/c | n/c | n/c | n/c |
|  | all | 0.89±0.21 | 0.60 | 0.57±0.03 | 0.52 | 6 | 0.97±0.23 | 0.50 | 0.61±0.27 | 0.39 | 17 |
| BS | fl 3 | 11.77±2 | 8.66 | 1.53±0.21 | 1.17 | 7 | - | - | - | - | - |
| PS | fl 3 | 28.25±3.44 | 22.21 | 1.87±0.19 | 1.59 | 11 | - | - | - | - | - |
| PS, tip | fl 3 | 6.83±1.86 | 4.24 | 1.88±0.19 | 1.59 | 8 | - | - | - | - | - |
| MPS | fl 3 | 25.74±2.29 | 22.46 | 2.3±0.15 | 2.09 | 6 | 21.55±2.5 | 16.38 | 1.73±0.35 | 1.27 | 10 |
| MPS, tip | fl 3 | 1.8±1.16 | 0.39 | 2.32±0.17 | 2.09 | 4 | 2.41±1.11 | 1.13 | 1.73±0.35 | 1.27 | 10 |

| Sensillum type | Localisation | *Megaphragma mymaripenne* | | | | |
| --- | --- | --- | --- | --- | --- | --- |
|  |  | Female | | | | |
|  |  | Length | Min length | Diameter | Min diameter | n |
| ChS-AP | sc | 14.25±1.72 | 11.82 | 0.8±0.07 | 0.68 | 14 |
|  | pd | 13.65±1.75 | 10.29 | 1.44±3.39 | 0.54 | 35 |
|  | fl 1 | 15.33±2.46 | 10.65 | 1.48±2.25 | 0.72 | 16 |
|  | all | 14.2±2.03 | 10.29 | 1.3±2.68 | 0.54 | 65 |
| TS1-AP | fl 1 | 10.72±2.03 | 7.01 | 0.78±0.11 | 0.55 | 67 |
|  | fl 2 | 13.38±3.64 | 6.68 | 0.84±0.13 | 0.56 | 199 |
|  | fl 3 | 19.8±5.42 | 5.15 | 0.98±0.15 | 0.68 | 42 |
|  | all | 13.68±4.52 | 5.15 | 0.85±0.14 | 0.55 | 308 |
| TS-UP | fl 2 | 29.93±4.78 | 21.84 | 1.36±0.19 | 0.75 | 32 |
|  | fl 3 | - | - | - | - | - |
|  | all | 29.93±4.78 | 21.84 | 1.36±0.19 | 0.75 | 32 |
| SS | fl 2 | 6.3±1.78 | 4.48 | 0.87±0.1 | 0.62 | 22 |
|  | fl 3 | 5.34±1.04 | 3.37 | 0.85±0.25 | 0.53 | 27 |
|  | all | 5.78±1.48 | 4.48 | 0.86±0.2 | 0.53 | 49 |
| TS2-AP | sc | 1.1±0.26 | 0.75 | 0.51±0.06 | 0.46 | 10 |
|  | pd | 0.79±0.08 | 0.76 | 0.51±0.08 | 0.35 | 12 |
|  | all | 0.93±0.24 | 0.75 | 0.51±0.07 | 0.35 | 22 |
| BS | fl 3 | 14.8±2.19 | 8.14 | 1.85±0.29 | 0.76 | 31 |
| PS | fl 3 | 29.77±3.84 | 18.37 | 2.22±0.51 | 0.83 | 49 |
| PS, tip | fl 3 | 9.17±4.08 | 3.46 | 2.22±0.42 | 1.57 | 62 |
| MPS | fl 3 | 31.94±3.57 | 21.69 | 2.45±0.5 | 1.57 | 29 |
| MPS, tip | fl 3 | 6.66±4.77 | 2.65 | 2.46±0.49 | 1.57 | 31 |
