# Supplementary material for: Between extreme simplification and ideal optimization: antennal sensilla morphology of miniaturized Megaphragma wasps (Hymenoptera: Trichogrammatidae)
Source: PeerJ. 2018 Nov 30;6:e6005. doi: 10.7717/peerj.6005 (PMC6276593; doi:10.7717/peerj.6005)
Supplement: Table S4 [file peerj-06-6005-s004.docx]

**Table S4.** References to the data used in regression analysis

| **Number of antennal sensilla in parasitoid wasps** | | | | |
| --- | --- | --- | --- | --- |
| **references** | | | **Number of antennal sensilla** | |
|  |  |  | **female** | **male** |
| *Trichogramma australicum* | Trichogrammatidae | (Amornsak, Cribb, and Gordh 1998) | 259 | - |
| *Anaphes victus* | Mymaridae | (Baaren, Boivin and Jean-pierre 1999) | 550 | - |
| *Anaphes listronoti* | Mymaridae | (Baaren, Boivin and Jean-pierre 1999) | 550 | - |
| *Microplitis croceipes* | Braconidae | (Das et al. 2011) | 7000 | 9000 |
| *Cotesia marginiventris* | Braconidae | (Das et al. 2011) | 6000 | 5000 |
| *Pteromalus puparum* | Pteromalidae | (Dweck 2009) | 1034 | - |
| *Microplitis pallidipes* | Braconidae | (Gao, Luo, and Hammond 2007) | 2660 | 2900 |
| *Encarsia guadeloupae* | Aphelinidae | (H. Zhou et al. 2013) | 522 | - |
| *Metaphycus parasaissetiae* | Encyrtidae | (Hou et al. 2013) | 850 | 620 |
| *Pteromalus cerealellae* | Pteromalidae | (Onagbola and Fadamiro 2008) | 1076 | 1115 |
| *Quadrastichus erythrinae* | Eulophidae | (Wild et al. 2013) | 194 | 178 |
| *Lysiphlebus fabarum* | Braconidae | (Xi et al. 2010) | 1237 | 1086 |
| *Ooencyrtus phongi* | Encyrtidae | (Xi et al. 2011) | 1775 | 497 |
| **Number of antennal sensilla types in parasitoid wasps** | | | |  |
| **references** | | | **Number of antennal sensilla types** | |
|  |  |  | **female** | **male** |
| *Trichogramma australicum* | Trichogrammatidae | (Amornsak, Cribb, and Gordh 1998) | 14 | 12 |
| *Apoanagyrus lopezi* | Encyrtidae | (Baaren, Barbier, and Nenon 1996) | 7 | - |
| *Leptomastix dactylopii* | Encyrtidae | (Baaren, Barbier, and Nenon 1996) | 7 | - |
| *Anaphes victus* | Mymaridae | (Baaren, Boivin and Jean-pierre 1999) | 7 | 4 |
| *Anaphes listronoti* | Mymaridae | (Baaren, Boivin and Jean-pierre 1999) | 7 | 4 |
| *Tetrastichus hagenowii* | EuIophidae | (Barlin, Vinson, and Piper 1981) | 6 | 5 |
| *Cotesia glomerata* | Braconidae | (Bleeker et al. 2004) | 6 | 6 |
| *Cotesia rubecula* | Braconidae | (Bleeker et al. 2004) | 6 | 6 |
| *Anagrus atomus* | Mymaridae | (Chiappini, Solinas, and Solinas 2001) | 7 | - |
| *Trichogramma galloi* | Trichogrammatidae | (Consoli, Kitajima and Parra 1999) | 7 | - |
| *Trichogramma pretiosum* | Trichogrammatidae | (Consoli, Kitajima and Parra 1999) | 7 | - |
| *Microplitis croceipes* | Braconidae | (Das et al. 2011) | 6 | 6 |
| *Cotesia marginiventris* | Braconidae | (Das et al. 2011) | 6 | - |
| *Pteromalus puparum* | Pteromalidae | (Dweck 2009) | 8 | - |
| *Microplitis pallidipes* | Braconidae | (Gao, Luo, and Hammond 2007) | 6 | - |
| *Encarsia guadeloupae* | Aphelinidae | (H. Zhou et al. 2013) | 8 | - |
| *Apanteles cypris* | Braconidae | (H. Zhou, Wu, Zhnag, & Zhnag, 2011) | 11 | 11 |
| *Metaphycus parasaissetiae* | Encyrtidae | (Hou et al. 2013) | 12 | 11 |
| *Theocolax elegans* | Pteromalidae | (Namikawa and Amornsak 2016) | 8 | 7 |
| *Pteromalus cerealellae* | Pteromalidae | (Onagbola and Fadamiro 2008) | 8 | 8 |
| *Macrocentrus cingulum* | Braconidae | (Wang 2013) | 9 | 9 |
| *Quadrastichus erythrinae* | Eulophidae | (Wild et al. 2013) | 6 | 6 |
| *Lysiphlebus fabarum* | Braconidae | (Xi et al. 2010) | 7 | 7 |
| *Ooencyrtus phongi* | Encyrtidae | (Xi et al. 2011) | 10 | 7 |
| *Trichogramma dendrolimi* | Trichogrammatidae | (Zhang et al. 2012) | 13 | 10 |

| **Sizes of antennal sensilla in parasitoid wasps** | | |
| --- | --- | --- |
| *Anaphes listronoti* | Mymaridae | (Baaren, Boivin and Jean-pierre 1999) |
| *Anaphes victus* | Mymaridae | (Baaren, Boivin and Jean-pierre 1999) |
| *Cotesia rubecula* | Braconidae | (Bleeker et al. 2004) |
| *Trichogramma pretiosum* | Trichogrammatidae | (Consoli, Kitajima and Parra 1999) |
| *Trichogramma galloi* | Trichogrammatidae | (Consoli, Kitajima and Parra 1999) |
| *Microplitis croceipes* | Braconidae | (Das et al. 2011) |
| *Cotesia marginiventris* | Braconidae | (Das et al. 2011) |
| *Apanteles cypris* | Braconidae | (H. Zhou, Wu, Zhnag, & Zhnag, 2011) |
| *Metaphycus parasaissetiae* | Encyrtidae | (Hou et al. 2013) |
| *Theocolax elegans* | Pteromalidae | (Namikawa and Amornsak 2016) |
| *Pteromalus cerealellae* | Pteromalidae | (Onagbola and Fadamiro 2008) |
| *Trissolcus japonicus* | Scelionidae | (Shi-yong et al. 2015) |
| *Trissolcus plautiae* | Scelionidae | (Shi-yong et al. 2015) |
| *Macrocentrus cingulum* | Braconidae | (Wang 2013) |
| *Quadrastichus erythrinae* | Eulophidae | (Wild et al. 2013) |
| *Trichogramma evanescens* | Trichogrammatidae | (Woude and Smid 2015) |
| *Lysiphlebus fabarum* | Braconidae | (Xi et al. 2010) |
| *Ooencyrtus phongi* | Encyrtidae | (Xi et al. 2011) |
| *Trichogramma dendrolimi* | Trichogrammatidae | (Zhang et al. 2012) |
