# Supplementary material for: Between extreme simplification and ideal optimization: antennal sensilla morphology of miniaturized Megaphragma wasps (Hymenoptera: Trichogrammatidae)
Source: PeerJ. 2018 Nov 30;6:e6005. doi: 10.7717/peerj.6005 (PMC6276593; doi:10.7717/peerj.6005)
Supplement: Table S5 [file peerj-06-6005-s005.docx]

**Table S5.** Comparison of the terminology adopted in this work with the terminology of some of the key works on parasitoid wasps antennal sensilla.

|  | **Morphological sensillum types** | | | | |
| --- | --- | --- | --- | --- | --- |
|  | **ChS-AP** | **TS1-AP** | **TS2-AP** | **BS** | **MPS** |
| Das et al., 2011 | ST | - | - | SB 1 | PL |
| Baaren et al., 1999 | TS | - | - | SC 2 | PS |
| Zhou et al., 2013b | TS-1 | - | CH-1. CH-2 | BS-4 | PS |
| Chiappini, Solinas & Solinas, 2001 | TS | - | - | AS | SR |
| Zhang et al., 2012 | ChS | TS 3 | - | - | PS |
| Olson & Andow, 1993 | AP trichodea B | AP trichodea A | - | - | MPP placodea A |
| Amornsak, Cribb & Gordh, 1998 | ChS 1 | TS 2 | - | - | PS |
| Dweck, 2009 | ST1-NP | ST2-NP | Sc-NP | - | MSP |
| Xi et al., 2010 | ST | SCh 1 | SC II | - | SP |
| Onagbola & Fadamiro, 2008 | ST2-AP | - | ST4-AP | - | MPS |
| Woude & Smid, 2015 | ATS | SS | - | - | MPS |
